# When Do Scanograms and Standing Ankle Radiographs Differ? A Comparative Analysis of Ankle Alignment Measurements

**DOI:** 10.3390/jcm15176553

**Published:** 2026-08-25

**Authors:** In Uk Yeo, Tae Gyun Kim, Byung Ki Cho, Young Ki Min, Hyung Gyu Cho, Chang-Geun Yu, Jae Hwang Song

**Affiliations:** 1 Department of Orthopaedic Surgery, Konyang University Hospital, Daejeon 35365, Republic of Korea; dudlsdnr@naver.com (I.U.Y.); ktk1113@kyuh.ac.kr (T.G.K.); myk48141408@gmail.com (Y.K.M.); chg6391@kyuh.ac.kr (H.G.C.); h761@naver.com (C.-G.Y.); 2 Department of Orthopaedic Surgery, College of Medicine, Konyang University, Daejeon 35365, Republic of Korea; 3 Department of Orthopaedic Surgery, Chungbuk National University Hospital, College of Medicine, Chungbuk National University, Cheongju 28644, Republic of Korea; titanick25@naver.com

**Keywords:** ankle osteoarthritis, lower extremity scanogram, standing ankle radiograph, Takakura classification, ankle alignment

## Abstract

**Background:** Lower extremity scanograms are widely used to evaluate lower limb alignment and are frequently used as a substitute for standing ankle radiographs in retrospective studies. However, their reliability for evaluating ankle osteoarthritis remains unclear. This study investigated radiographic discrepancies between lower extremity scanograms and standing ankle radiographs and identified the patient populations in whom scanograms may not be an appropriate alternative. **Methods:** This retrospective study included 90 ankles that underwent both lower extremity scanograms and standing ankle radiographs. Based on the agreement of Takakura staging between the two imaging modalities, ankles were classified into a non-discrepancy group (NDG), discrepancy group (DG), or inadequate visualization group (IVG). Radiographic parameters were compared between groups and between imaging modalities. **Results:** Patients in the IVG had significantly lower Takakura stages than those in the NDG + DG group on standing ankle radiographs (*p* = 0.013), suggesting that inadequate visualization was more frequently associated with early-stage ankle osteoarthritis. Talar tilt angle (TT) measured on scanograms (*p* = 0.008) and tibial anterior surface angle (TAS) measured on both scanograms (*p* = 0.013) and standing ankle radiographs (*p* = 0.005) also differed significantly between the groups. Compared with the DG, the NDG demonstrated significantly higher Takakura stages on scanograms (*p* = 0.012). Within-patient comparisons revealed statistically significant differences in TT, TAS, and tibial plafond inclination (TPI) between the two imaging modalities; however, the clinical relevance of these differences remains uncertain because validated thresholds for clinically meaningful differences between the two imaging techniques have not been established. Staging discrepancies were significantly more common in early-stage (Takakura stages 1–3a) than advanced-stage (stages 3b–4) ankle osteoarthritis (*p* = 0.010). **Conclusions:** In this retrospective, single-center study of 90 ankles, lower extremity scanograms showed discrepancies from standing ankle radiographs in the evaluation of ankle osteoarthritis, particularly in early-stage disease. These findings suggest that scanograms may not provide equivalent ankle-specific information in all patients. Standing ankle radiographs may be considered when precise ankle-specific assessment is clinically important, particularly in patients with suspected early-stage ankle osteoarthritis.

## 1. Introduction

The prevalence of knee and ankle osteoarthritis has been increasing with the extension of life expectancy and the growing participation of the aging population in sports and physical activities [1]. To properly evaluate and treat arthritis of the lower extremity joints, whether in the knee or ankle, it is important to assess the overall alignment of the hip, knee, and ankle together [2].

Weight-bearing ankle radiographs play a central role in the evaluation of ankle osteoarthritis because radiographic staging and treatment planning depend largely on the distribution and severity of tibiotalar joint-space narrowing and coronal alignment [3]. In particular, the Takakura classification is based on progressive medial joint-space narrowing and obliteration on weight-bearing radiographs. Therefore, differences in beam centering or projection may affect the apparent joint space and potentially alter disease staging. This issue is clinically relevant because lower extremity scanograms are increasingly used to evaluate ankle alignment in studies of lower-limb realignment procedures, even though these images are primarily designed to assess global lower-extremity alignment rather than the ankle joint itself [4,5,6]. Global lower-extremity alignment and local ankle-joint morphology therefore represent related but distinct radiographic targets. Full-length lower-extremity imaging is primarily intended to characterize mechanical-axis relationships across the hip, knee, and ankle, whereas dedicated weight-bearing ankle radiographs are centered on the tibiotalar joint and are better suited for evaluating local joint-space narrowing and ankle-specific coronal deformity [2,3]. Consequently, an imaging modality suitable for global lower-extremity alignment assessment may not necessarily provide equivalent information for the evaluation and staging of ankle osteoarthritis.

However, in retrospective studies evaluating perioperative changes in lower extremity alignment after knee surgery, analysis may be limited in patients who did not undergo standing ankle radiograph. In particular, recent studies investigating changes in ankle alignment or degeneration following knee osteoarthritis procedures, such as high tibial osteotomy (HTO) and total knee replacement (TKR), have frequently relied on lower extremity scanograms because standing ankle radiographs were unavailable in many cases [4,5,6].

The authors experienced several cases in which radiographic parameters of the ankle differed between standing ankle radiographs and lower extremity scanograms. Furthermore, a few ankle parameters were not measurable on one of the imaging modalities in some cases. These observations prompted us to investigate the patient characteristics associated with such discrepancies and to evaluate the differences between the two imaging modalities.

To date, no study has investigated which patients demonstrate discrepancies in specific ankle parameters between standing ankle radiographs and scanograms, or which ankle parameters cannot be reliably measured on certain imaging modalities. Therefore, we conducted this study under the hypothesis that significant differences exist between standing ankle radiographs and lower extremity scanograms in selected patients, and that some ankle parameters may not be reliably assessed depending on the imaging modality used. The purpose of this study was to identify the patient populations in whom scanograms should not be used as a substitute for standing ankle radiographs for ankle joint evaluation.

## 2. Materials and Methods

### 2.1. Patient Enrollment

This retrospective single-center study was conducted after approval from the Institutional Review Board of our institution (Konyang University Hospital, IRB No.: KYUH 2025 11 004). The written consent from participants was not needed due to the retrospective nature of the study.

Patients were eligible for inclusion if they (1) presented with ankle pain and (2) underwent both weight-bearing lower extremity scanography and standing ankle radiography during the study period [6,7,8,9]. All included patients were evaluated primarily for ankle symptoms, and the scanograms were obtained as part of the assessment of overall lower-extremity alignment in patients with ankle pain. Since varus ankle osteoarthritis is the most common pattern of ankle osteoarthritis and the Takakura classification is primarily applicable to varus rather than valgus ankle osteoarthritis, we considered the exclusion of valgus ankle osteoarthritis to be more appropriate for the present study design. In addition, we excluded patients with congenital deformities or a history of previous hip, knee, or ankle surgery because these conditions could independently affect lower-extremity alignment and potentially confound the study outcomes. Accordingly, among 140 ankles from 70 patients who presented with ankle pain and underwent both lower extremity scanogram and standing ankle radiograph at our institution, valgus ankle osteoarthritis (*n* = 13) and congenital deformities (*n* = 10) or a previous history of hip, knee, or ankle surgery (*n* = 27) were excluded. Consequently, a total of 90 ankles were included in the final analysis.

**Figure 1:**
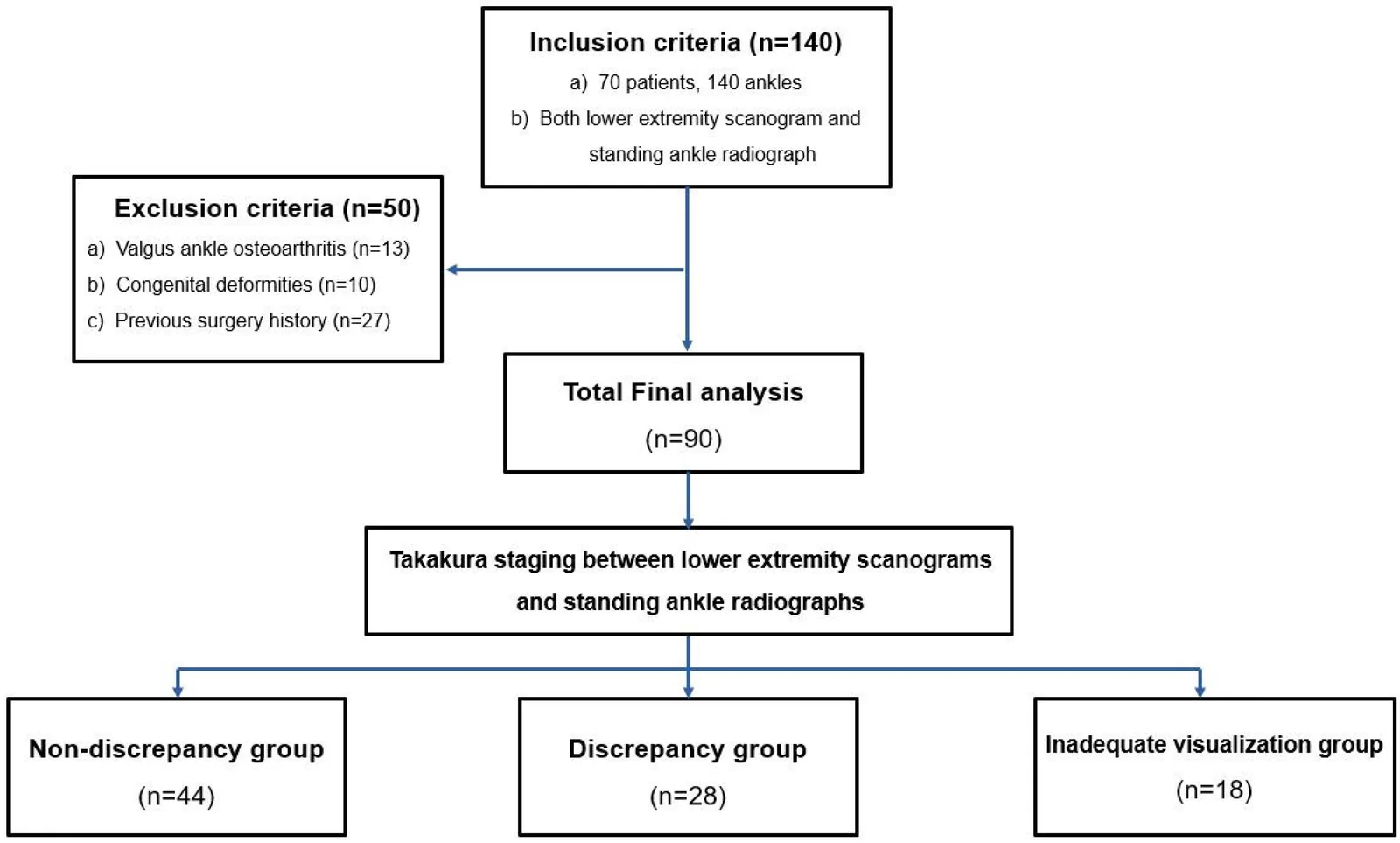
Flow diagram of participant recruitment and group allocation. Patients were classified according to the agreement of Takakura staging between lower extremity scanograms and standing ankle radiographs. The non-discrepancy group (NDG) included ankles with identical Takakura stages on both imaging modalities; the discrepancy group (DG) included ankles with different Takakura stages between the two modalities; and the inadequate visualization group (IVG) included ankles in which accurate Takakura staging could not be determined on one of the two imaging modalities because of inadequate radiographic visualization.

**Figure 2:**
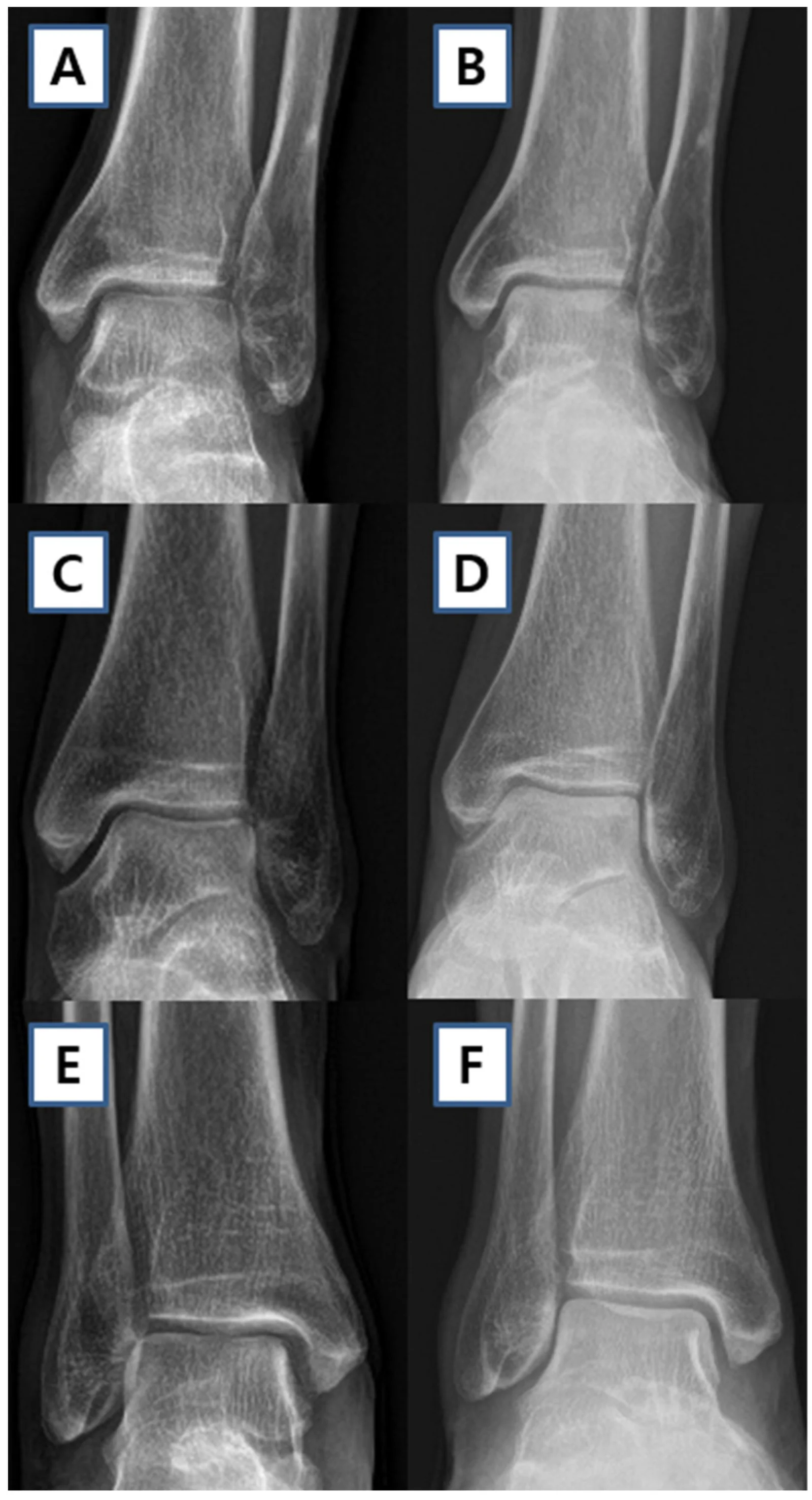
Representative radiographs of the three study groups. (**A**,**B**) Non-discrepancy group (NDG): Takakura staging was identical (stage 1) on the lower extremity scanogram (**A**) and standing ankle radiograph (**B**). (**C**,**D**) Discrepancy group (DG): the ankle was classified as Takakura stage 1 on the lower extremity scanogram (**C**) but as stage 3a on the standing ankle radiograph (**D**). (**E**,**F**) Inadequate visualization group (IVG): accurate Takakura staging could not be performed on the lower extremity scanogram (**E**) because the tibiotalar joint and tibial plafond were inadequately visualized, whereas the standing ankle radiograph (**F**) allowed appropriate assessment and was classified as Takakura stage 1.

Takakura staging was independently evaluated on both lower extremity scanograms and standing ankle radiographs [7]. Based on the agreement between the lower extremity scanogram and standing ankle radiograph, the ankles were categorized into three groups: Non-discrepancy group (NDG), in which the Takakura stage was identical on both imaging studies; Discrepancy group (DG), in which discrepant Takakura stages were identified between the two modalities; and Inadequate visualization group (IVG), in which accurate Takakura staging could not be determined on one of the imaging modalities (either the scanogram or standing ankle radiograph) because of radiographic limitations. Subsequently, comparative analyses were performed between the combined NDG + DG cohort vs. IVG, as well as between NDG vs. DG (Figure 1 and Figure 2).

Among all patients in whom Takakura staging was feasible (NDG and DG), patients were further divided into a lower-stage group (Group 1) and a higher-stage group (Group 2) according to the severity of Takakura stage. Given that the transition from Stage 3a to Stage 3b represents a critical threshold beyond which surgical treatment strategies differ substantially, Stage 3b was adopted as the cutoff to distinguish between the two groups [8,9].

The demographic data including sex, age, laterality, and medical history of the participants were evaluated by reviewing the patients’ electronic medical records. Medical records were reviewed by an investigator who was not involved in the diagnosis or treatment of the patients.

### 2.2. Radiographic Evaluation

All radiographic parameters were measured using standing anteroposterior and lateral ankle radiographs and full-length lower extremity scanograms. All measurements were obtained digitally through the institutional Picture Archiving Communication System (It) software.

### 2.3. Radiographic Acquisition Techniques for Lower Extremity Scanograms and Standing Ankle Radiographs

**Figure 3:**
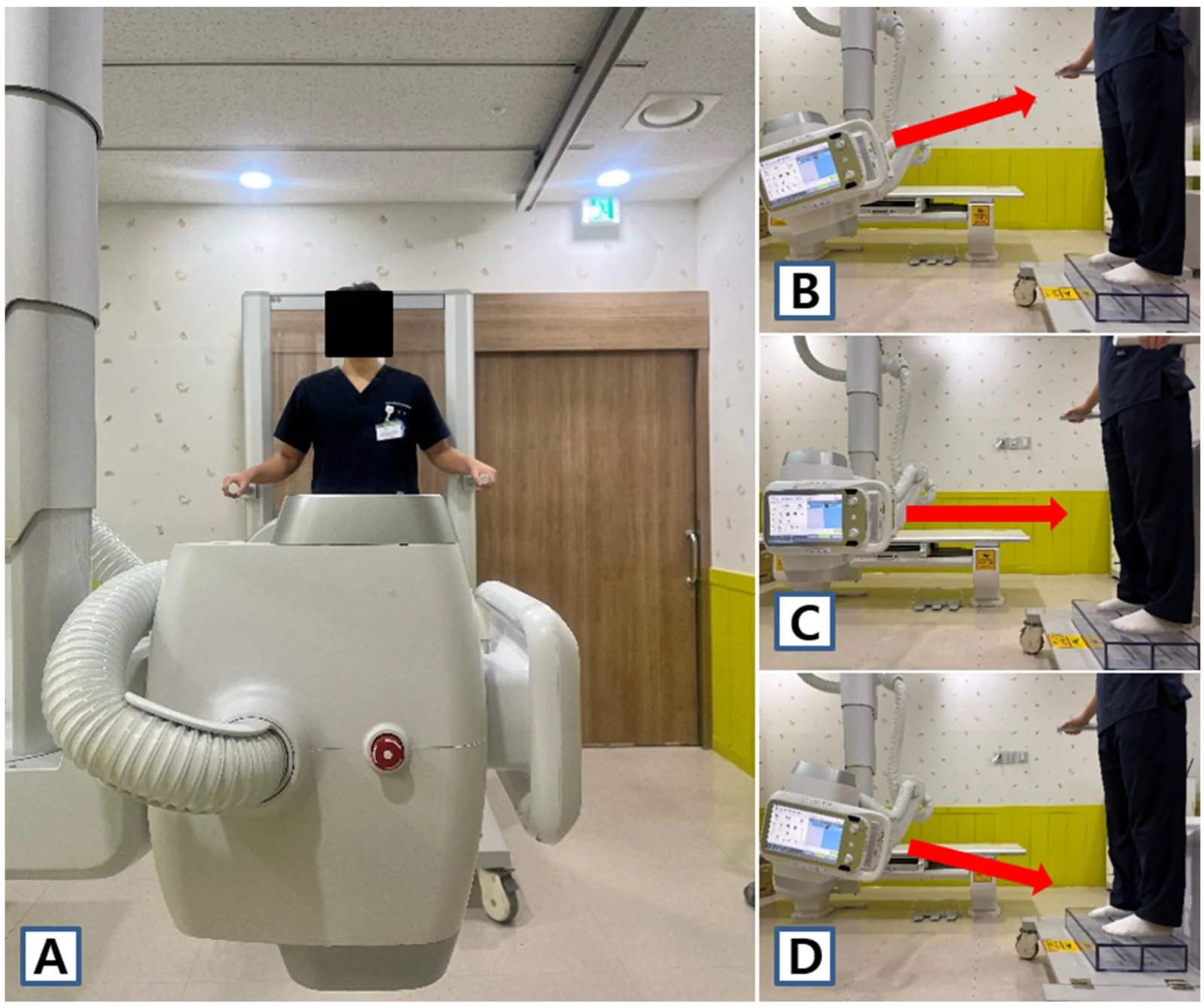
Lower extremity scanogram radiographic acquisition techniques. (**A**) shows the frontal position of the X-ray beam projector during scanogram acquisition. (**B**–**D**) show the lateral position and angulation of the beam projector during hip, knee, and ankle imaging, respectively. Red arrows in (**B**–**D**) indicate the direction of the X-ray beam.

Lower extremity scanograms were obtained under weight-bearing conditions to evaluate overall lower limb alignment. Patients were positioned in a standing posture with both lower extremities bearing weight simultaneously. The X-ray tube (Samsung GC85A, Suwon-si, Republic of Korea) was initially centered at the knee level, with a source-to-image distance of 190 cm. The tube was subsequently angled toward the hip and ankle joints to acquire the corresponding segments, which were combined into a full-length lower extremity image (Figure 3) [10]. The exact beam angulation varied according to individual patient anatomy and was not routinely recorded.

**Figure 4:**
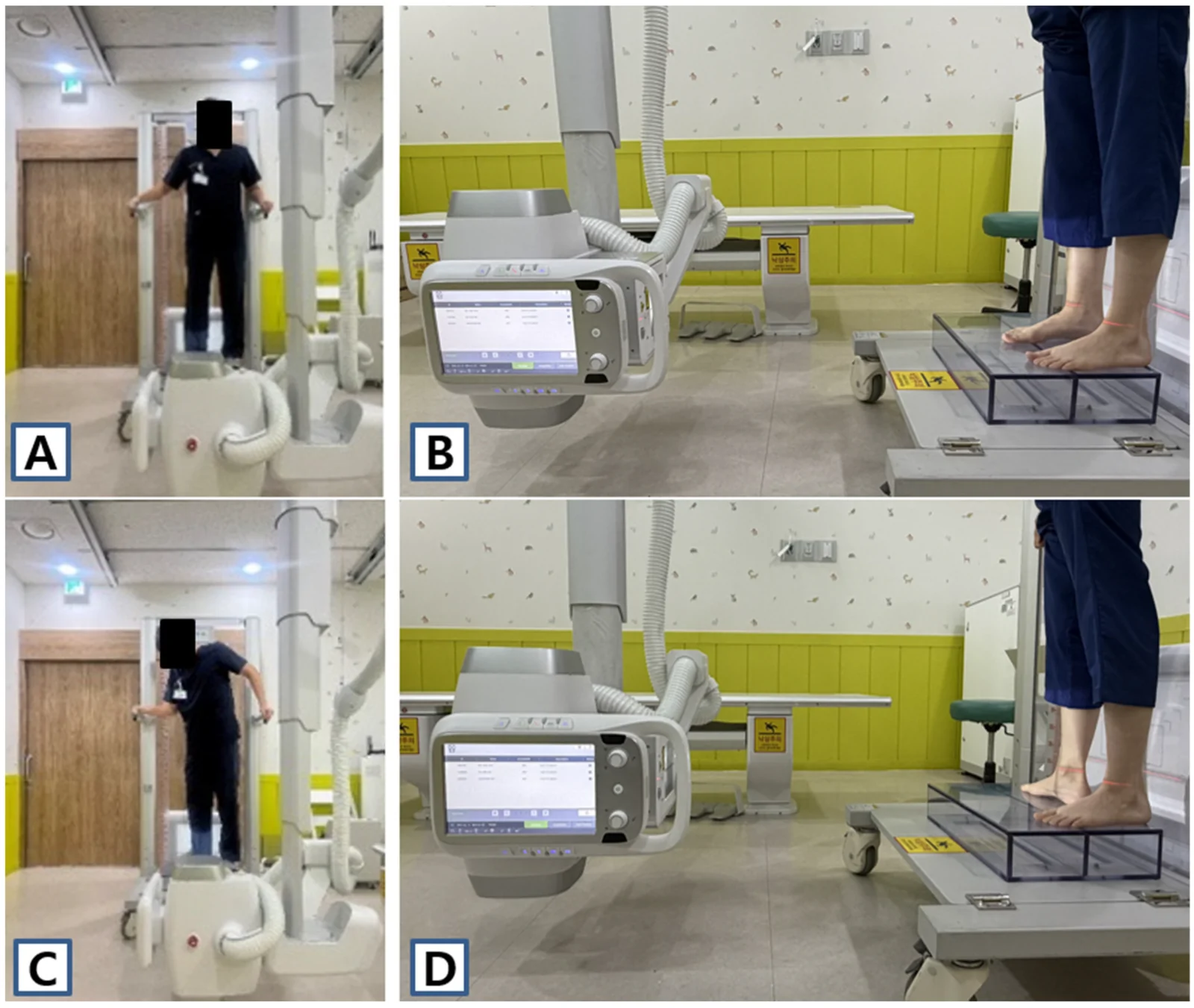
Standing ankle AP/Lat radiographic acquisition techniques. (**A**,**B**) demonstrate the frontal and lateral positions of the X-ray beam projector, respectively, during standing ankle AP radiograph. (**C**,**D**) show the frontal and lateral positions of the X-ray beam projector, respectively, during standing ankle lateral radiograph.

For standing ankle radiographs, each ankle was imaged separately in an upright weight-bearing position using the same radiographic system and a source-to-image distance of 190 cm. The X-ray tube was positioned at the level of the ankle joint, and the central beam was directed toward the ankle joint to obtain standard weight-bearing ankle radiographs (Figure 4) [11].

### 2.4. Radiographic Assessment in Lower Extremity Scanogram and Standing Ankle X-Ray

Radiographic parameters that could be evaluated on both standing ankle radiographs and lower extremity scanograms included the Takakura stage, talar tilt angle (TT), tibial anterior surface angle (TAS), tibial plafond inclination (TPI), talar inclination (TI), and medial clear space distance (MCS). All radiographic measurements were performed according to previously established and validated methods [6,12,13,14,15,16]. The degree of ankle osteoarthritis was categorized according to the Takakura classification based on weight-bearing anteroposterior ankle radiographs. Stage 1 indicated early degenerative change with osteophyte formation or subchondral sclerosis without apparent narrowing of the joint space. Stage 2 was defined by narrowing localized to the medial side of the tibiotalar joint. Stage 3a represented complete loss of the medial joint space extending to the level of the medial malleolus. Stage 3b referred to obliteration of the joint space progressing toward the superior aspect of the talar dome. Stage 4 demonstrated diffuse degeneration with complete disappearance of the tibiotalar joint space [7].

Parameters evaluable solely on standing ankle lateral radiographs included the tibial lateral surface angle (TLS), which allowed assessment of sagittal plane orientation of the distal tibial articular surface.

**Figure 5:**
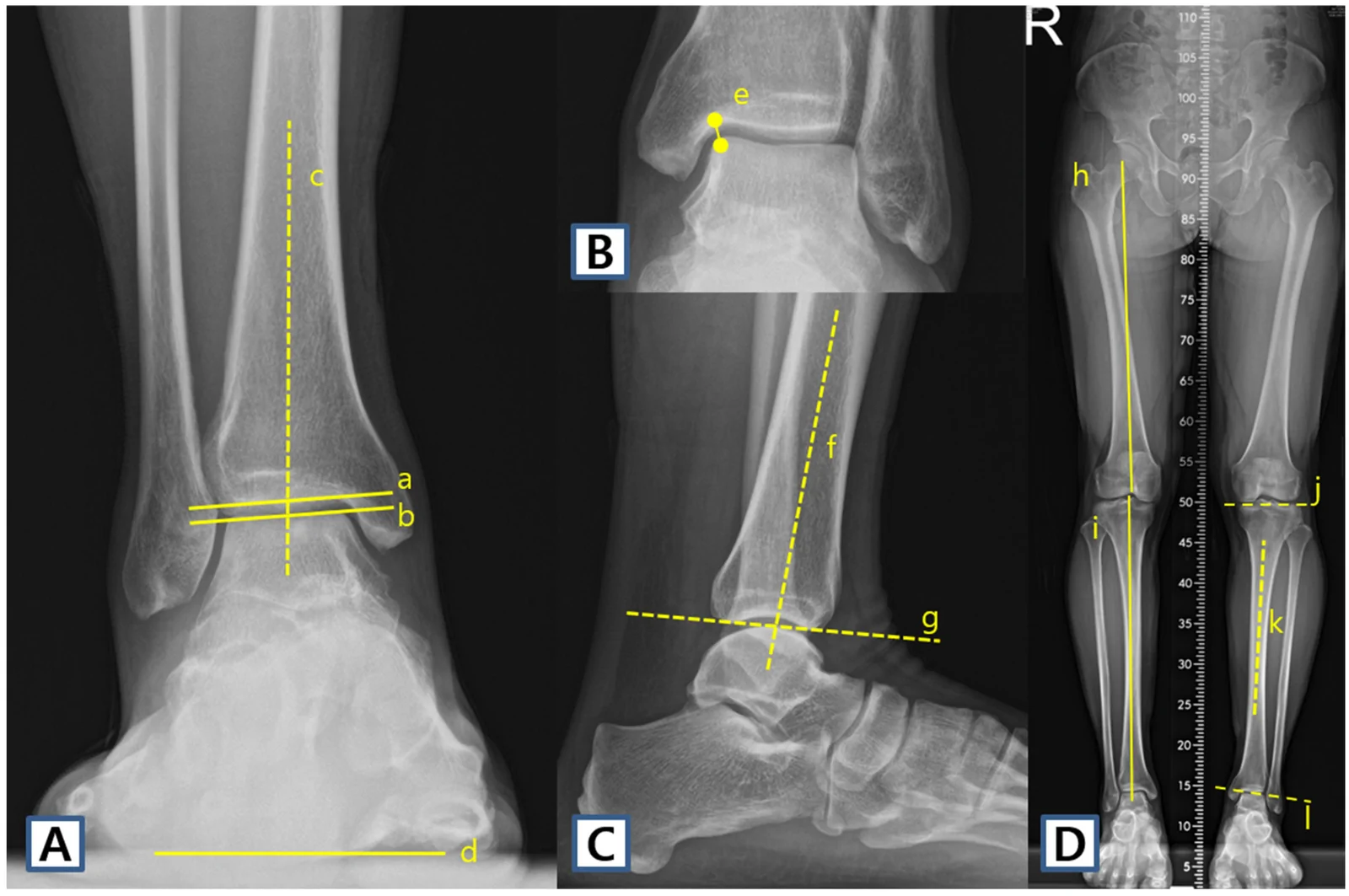
Radiologic parameters in lower extremity scanogram and standing Ankle X-ray: (**A**) Talar tilt angle (a and b), tibial anterior surface angle (a and c), tibial plafond inclination (a and d), talar inclination (b and d). (**B**) The medial clear space (e) was defined as the shortest distance between the lateral border of the medial malleolus and the medial border of the talus at the level of the ankle mortise. (**C**) Tibial lateral surface angle (f and g) was evaluated in the ankle lateral radiograph. (**D**) Hip-knee-ankle angle (h and i), medial proximal tibial angle (j and k), knee-tibial plafond angle (j and l) was measured on full-length standing lower extremity scanograms. Leg length discrepancy was measured as the difference in the distance from the center of the femoral head (hip center) to the center of the talar dome between the two lower extremities.

Additional parameters assessable exclusively on standing lower extremity scanograms included the hip-knee-ankle angle (HKA angle), medial proximal tibial angle (MPTA), knee-tibial plafond angle (KTPA), and leg length discrepancy (LLD), which collectively provided information regarding overall coronal alignment and limb length symmetry of the lower extremity (Figure 5).

Two orthopaedic surgeons with more than 10 and 5 years of clinical experience, respectively, independently reviewed all radiographs. In cases of disagreement between the two observers, the final Takakura stage used for group allocation was determined by consensus.

### 2.5. Statistics Analysis

SPSS version 22 (IBM, Armonk, NY, USA) was used for statistical analyses. Normality was assessed using the Shapiro–Wilk test. For comparisons between two independent groups, normally distributed continuous variables were analyzed using the independent-samples *t*-test, whereas non-normally distributed variables were analyzed using the Mann–Whitney U test. Categorical variables were analyzed using the chi-squared test or Fisher’s exact test, as appropriate. For comparisons of paired measurements obtained from the same patients using the two imaging modalities, the normality of the within-patient differences was assessed. Normally distributed paired differences were analyzed using the paired *t*-test, whereas non-normally distributed paired differences were analyzed using the Wilcoxon signed-rank test. Takakura stages, as ordinal variables, were analyzed using nonparametric tests.

Interobserver agreement for Takakura staging was assessed using Cohen’s kappa coefficients. Interobserver reliability for continuous radiographic parameters was evaluated using intraclass correlation coefficients (ICCs) using a two-way random-effects model with absolute agreement for single measurements.

## 3. Results

No significant differences in demographic characteristics were observed among NDG + DG vs. IVG (Table 1). Interobserver agreement for Takakura staging was almost perfect (κ > 0.81) on both scanograms and standing ankle radiographs (Supplementary Table S1). Interobserver reliability for other radiologic parameters was good to excellent across both imaging modalities, with ICCs > 0.75.

Comparisons between NDG + DG and IVG revealed significant differences in Takakura stage measured on standing ankle radiographs (*p* = 0.013), TT measured on scanograms (*p* = 0.008), and TAS measured on both scanograms (*p* = 0.013) and standing ankle radiographs (*p* = 0.005) (Table 2).

A significant difference in Takakura stage was observed between NDG and DG (*p* = 0.005) (Table 3).

Comparisons between NDG and DG demonstrated a significant difference in Takakura stage measured on scanograms (*p* = 0.012). Within NDG, significant differences between scanogram and standing ankle radiograph measurements were identified for TT (*p* = 0.004), TAS (*p* < 0.001), and TPI (*p* = 0.012). Within DG, significant differences were observed for Takakura stage (*p* = 0.001), TAS (*p* = 0.004), and TPI (*p* = 0.022) (Table 4).

The proportion of concordant and discordant staging differed significantly between Group 1 (Takakura stage 1–3a group) and Group 2 (Takakura stage 3b–4) (*p* = 0.010) (Table 5).

## 4. Discussion

The present study was initiated based on the hypothesis that lower extremity scanograms may have limitations in evaluating ankle osteoarthritis because the X-ray beam is projected from different positions compared with standing ankle radiographs. After reviewing 90 ankles, three major findings were identified. First, discrepancies in Takakura staging between the two imaging modalities were more frequently observed in patients with mild ankle osteoarthritis. Second, Takakura staging could not be performed because of radiographic limitations, particularly in patients with mild osteoarthritis. Third, statistically significant differences were observed in selected radiographic parameters, particularly TT, TAS, and TPI, between lower extremity scanograms and standing ankle radiographs obtained from the same patients. However, these statistical differences should not be assumed to represent clinically meaningful differences.

In the comparison between patients in whom all radiographic parameters, including Takakura stage, could be evaluated on both imaging modalities (NDG + DG) and those in whom evaluation was not feasible (IVG), standing ankle radiographs demonstrated a significant difference in Takakura stage between the two groups: the mean Takakura stage was significantly higher in the NDG + DG group than in the IVG group. These findings suggest that advanced ankle osteoarthritis may allow more reliable assessment on both imaging modalities. Most patients classified into the IVG had inadequate visualization of the tibial plafond on at least one of the two imaging modalities. Previous studies have demonstrated that ankle positioning and X-ray beam projection angle can substantially influence the apparent morphology and radiographic measurements of the ankle. Therefore, in patients with mild osteoarthritis, differences in beam orientation may lead to oblique projection or superimposition of the tibial plafond and tibiotalar joint space, potentially obscuring subtle joint-space narrowing [17,18].

Significant differences were also observed in TT and TAS in Table 2 (NDG + DG vs. IVG). As varus ankle osteoarthritis progresses, TT generally tends to increase, reflecting progressive coronal plane instability of the ankle joint [15]. Consistent with this, the mean TT was greater in the NDG + DG group than in the IVG group, further supporting the notion that advanced ankle osteoarthritis is associated with improved visualization and assessment on both imaging modalities. Similarly, the TAS demonstrated a significant difference on standing ankle radiographs, with mean values of 87.93° and 89.71° in the NDG + DG and IVG groups, respectively. These findings suggest that patients in the NDG and DG groups had more advanced ankle varus deformity, further supporting the above interpretation.

Comparison between the NDG and DG groups revealed that the mean Takakura stage on scanograms was significantly higher in the NDG group. We interpreted this finding as indicating that advanced ankle osteoarthritis is frequently associated with more severe joint space obliteration, resulting in consistent Takakura staging regardless of differences in X-ray beam projection. Based on this observation, an additional chi-square analysis was performed (Table 5), which demonstrated that Patients with mild ankle osteoarthritis (Group 1) constituted a significantly larger proportion of the DG, whereas no patients with severe ankle osteoarthritis (Group 2) were classified into the DG. These findings are consistent with the anatomical progression represented by the Takakura classification. In early-stage disease, subtle medial joint-space narrowing may be sensitive to small differences in beam projection, whereas in advanced stages, extensive joint-space obliteration is readily apparent despite modest differences in radiographic projection [7]. Collectively, these findings suggest that lower extremity scanograms may underestimate the severity of ankle osteoarthritis or even preclude accurate radiographic assessment in patients with early-stage disease.

The statistically significant within-patient differences in TT, TAS, and TPI observed in Table 4 may reflect differences in radiographic acquisition geometry between the two imaging modalities. One possible explanation is the difference in X-ray beam orientation between lower extremity scanograms and dedicated standing ankle radiographs [17,18]. In dedicated standing ankle radiographs, the central beam is directed toward the ankle joint, whereas scanogram acquisition requires sequential beam angulation from a tube initially centered at the knee level. This angulation may obscure the true tibiotalar joint space and create overlapping shadows. Consequently, the distal tibial plafond may be projected more obliquely on scanograms, potentially altering apparent joint-space width and angular measurements. This effect may be particularly relevant in early-stage ankle osteoarthritis, in which subtle joint-space narrowing is more susceptible to projectional variation. However, because the exact beam angulation during scanogram acquisition was not recorded, this proposed mechanism could not be directly tested in the present study and therefore remains hypothetical. Prospective studies using standardized and recorded beam geometry are required to verify this explanation. Although statistically significant differences were observed in several angular measurements, their clinical significance should be interpreted cautiously. Previous studies have proposed absolute radiographic thresholds for surgical planning in varus ankle osteoarthritis [19]; however, no validated threshold has been established for a clinically meaningful difference in TT, TAS, or TPI between different radiographic acquisition methods. Therefore, the statistically significant differences observed in the present study should not be interpreted as necessarily altering surgical indications or osteotomy planning. Further studies are needed to investigate these outcomes and determine their clinical significance.

To our knowledge, this is the first study to investigate radiographic discrepancies between lower extremity scanograms and standing ankle radiographs. Although lower extremity scanograms have frequently been used to assess the ankle parameters in patients undergoing lower limb realignment procedures, the limitations of this practice have not previously been addressed. Multiple radiographic parameters involving not only the ankle joint but also lower limb alignment were comprehensively evaluated, allowing assessment of overall radiographic trends.

From a broader imaging perspective, these findings also highlight the inherent dependence of two-dimensional radiographic measurements on patient positioning and X-ray projection. Weight-bearing computed tomography (WBCT) enables three-dimensional assessment of the ankle under physiological loading with less dependence on projectional variation and has increasingly been used to evaluate joint-space distribution, hindfoot alignment, and three-dimensional deformity in ankle osteoarthritis [20,21]. Future prospective studies using WBCT as a reference standard may therefore help determine the extent to which the discrepancies observed between scanograms and dedicated ankle radiographs reflect true differences in ankle alignment rather than projection-related effects.

Beyond the methodological implications of the present findings, accurate characterization of ankle and lower-extremity alignment may also have translational relevance for patient-specific deformity correction. In varus ankle deformity, weight-bearing CT-based three-dimensional planning and patient-specific guides have been applied to supramalleolar osteotomy, and 3D-printing-assisted techniques have demonstrated the feasibility of reproducing individualized correction plans [22,23]. More broadly, advances in additive manufacturing have enabled individualized orthopaedic implant design using parametric modeling and selective laser melting, while the mechanical properties of 3D-printed metallic components remain an important consideration for their clinical translation [24,25]. Accordingly, reliable modality-specific assessment of ankle alignment may become increasingly important as radiographic and three-dimensional imaging data are incorporated into personalized surgical planning and patient-specific guide or implant design.

This study has several limitations. First, its retrospective design may have introduced selection bias. Second, this was a single-center study with a relatively limited sample size, which may restrict the generalizability of the findings. Third, the exact X-ray beam angulation during scanogram acquisition was not recorded. Therefore, although differences in projection geometry may plausibly have contributed to the discrepancies observed between the two imaging modalities, this mechanism could not be directly evaluated and remains hypothetical. Fourth, although statistically significant differences in TT, TAS, and TPI were identified between the imaging modalities, no validated thresholds have been established for clinically meaningful intermodality differences in these parameters. Accordingly, the clinical significance of these differences remains uncertain. Future prospective, multicenter studies using standardized radiographic acquisition protocols are needed to confirm these findings.

## 5. Conclusions

Based on this retrospective, single-center study of 90 ankles, discrepancies were observed between lower extremity scanograms and standing ankle radiographs in the evaluation of ankle osteoarthritis, particularly in patients with early-stage disease. Lower extremity scanograms may underestimate disease severity or provide inadequate visualization in some patients. However, the clinical significance of the statistically significant differences in TT, TAS, and TPI remains uncertain because validated thresholds for clinically meaningful differences between these imaging techniques have not been established. Therefore, these findings should be interpreted cautiously. When precise ankle-specific assessment is clinically important, particularly in patients with suspected early-stage ankle osteoarthritis, dedicated standing ankle radiographs may be considered rather than relying solely on lower extremity scanograms. Prospective multicenter studies with standardized radiographic acquisition protocols are needed to confirm these observations.

## Figures and Tables

**Table 1:** Baseline demographic characteristics of NDG + DG vs. IVG.

Characteristics	NDG + DG (*n* = 72)	IVG (*n* = 18)	*p* Value
Sex			0.797
Male	35 (48.6)	8 (44.4)	
Female	37 (51.4)	10 (55.6)	
Age (years) ^a^	62.4	66.6	0.132
Ankle			0.063
Right	32 (44.4)	13 (72.2)	
Left	40 (55.6)	5 (27.8)	
Takakura stage			0.118
1	15 (20.8)	9 (50.0)	
2	21 (29.2)	5 (27.8)	
3a	27 (37.5)	3 (16.7)	
3b	3 (4.2)	0 (0.0)	
4	6 (8.3)	1 (5.6)	

Values are presented frequencies (%). ^a^ Values are presented as mean.

**Table 2:** Comparison of radiographic parameters between NDG + DG and IVG.

Characteristics	NDG + DG (*n* = 72)	IVG (*n* = 18)	*p* Value
Takakura stage			
Scanogram	2.12	1.83	0.318
Standing ankle X-ray	2.40	1.78	* 0.013
Talar tilt			
Scanogram	2.73	1.42	** 0.008
Standing ankle X-ray	1.89	0.98	0.094
Tibial anterior surface angle			
Scanogram	89.90	92.16	* 0.013
Standing ankle X-ray	87.93	89.71	** 0.005
Tibial plafond inclination			
Scanogram	3.17	3.37	0.886
Standing ankle X-ray	4.46	3.19	0.128
Talar inclination			
Scanogram	5.01	3.54	0.186
Standing ankle X-ray	6.73	3.53	0.253
Medial clear space			
Scanogram	2.78	3.21	0.237
Standing ankle X-ray	2.89	3.19	0.832
Tibial lateral surface angle	81.38	80.72	0.392
Hip-knee-ankle angle	2.00	2.49	0.538
Medial proximal tibial angle	85.87	86.55	0.901
Knee-tibial plafond angle	4.38	3.48	0.224
Leg length discrepancy	−1.19	−1.38	0.634

Values are presented as mean. * *p* < 0.05, ** *p* < 0.01. Values showing statistically significant differences are presented in bold.

**Table 3:** Baseline demographic characteristics of NDG and DG.

Characteristics	NDG (*n* = 44)	DG (*n* = 28)	*p* Value
Sex			0.235
Male	24 (54.5)	11 (39.3)	
Female	20 (45.5)	17 (60.7)	
Age (years) ^a^	62.9	61.7	0.963
Ankle			0.095
Right	16 (36.4)	16 (57.1)	
Left	28 (63.6)	12 (42.9)	
Takakura stage			** 0.005
1	13 (29.5)	2 (7.1)	
2	9 (20.5)	12 (42.9)	
3a	13 (29.5)	14 (50.0)	
3b	3 (6.8)	0 (0.0)	
4	6 (13.6)	0 (0.0)	
Mean	2.38	2.43	

Values are presented frequencies (%). ^a^ Values are presented as mean. ** *p* < 0.01. Values showing statistically significant differences are presented in bold.

**Table 4:** Comparison of radiographic parameters between NDG and DG.

Characteristics	NDG (*n* = 44)	DG (*n* = 28)	*p* Value
Takakura stage			
Scanogram	2.38	1.71	* 0.012
Standing ankle X-ray	2.38	2.43	0.947
*p* value ‡	1.000	** 0.001	
Talar tilt			
Scanogram	3.03	2.26	0.127
Standing ankle X-ray	1.99	1.73	0.685
*p* value ‡	** 0.004	0.368	
Tibial anterior surface angle			
Scanogram	89.98	89.78	0.860
Standing ankle X-ray	87.86	88.04	0.822
*p* value ‡	*** <0.001	** 0.004	
Tibial plafond inclination			
Scanogram	3.21	3.12	0.800
Standing ankle X-ray	4.42	4.53	0.977
*p* value ‡	* 0.012	* 0.022	
Talar inclination			
Scanogram	5.36	4.48	0.135
Standing ankle X-ray	8.00	4.76	0.680
*p* value ‡	0.094	0.552	
Medial clear space			
Scanogram	2.55	3.15	0.199
Standing ankle X-ray	2.64	3.28	0.060
*p* value ‡	0.838	0.707	
Tibial lateral surface angle	81.64	80.99	0.517
Hip-knee-ankle angle	2.12	1.82	0.704
Medial proximal tibial angle	85.80	85.96	0.670
Knee-tibial plafond angle	4.47	4.24	0.475
Leg length discrepancy	−0.65	−1.74	0.646

‡ *p* value between scanograom and standing ankle X-ray. Values are presented as mean. * *p* < 0.05, ** *p* < 0.01, *** *p* < 0.001. Values showing statistically significant differences are presented in bold.

**Table 5:** Comparison of staging concordance according to Takakura stage severity.

Characteristics	Group 1 Takakura Stage 1–3a (*n* = 63)	Group 2 Takakura Stage 3b–4 (*n* = 9)	*p* Value
Staging concordance			* 0.010
Same	35 (55.6%)	9 (100%)	
Different	28 (44.4%)	0 (0%)	

Values are presented as frequencies (%). * *p* < 0.05. Values showing statistically significant differences are presented in bold.

## Data Availability

The data presented in this study are available in this article.

## Supplementary Materials

The following supporting information can be downloaded at: https://www.mdpi.com/article/10.3390/jcm15176553/s1, Table S1: Interobserver agreement and reliability of radiographic assessments.
